# Targeting metabolism and AMP-activated kinase with metformin to sensitize non-small cell lung cancer (NSCLC) to cytotoxic therapy: translational biology and rationale for current clinical trials

**DOI:** 10.18632/oncotarget.17496

**Published:** 2017-04-27

**Authors:** Michael Troncone, Stephanie M. Cargnelli, Linda A. Villani, Naghmeh Isfahanian, Lindsay A. Broadfield, Laura Zychla, Jim Wright, Gregory Pond, Gregory R. Steinberg, Theodoros Tsakiridis

**Affiliations:** ^1^ Department of Oncology, McMaster University, Hamilton, Ontario, Canada; ^2^ Department of Biochemistry, Hamilton, Ontario, Canada; ^3^ Department of Medicine, Hamilton, Ontario, Canada; ^4^ Department of Pathology and Molecular Medicine, Hamilton, Ontario, Canada; ^5^ DeGroote School of Medicine, McMaster University, Hamilton, Ontario, Canada; ^6^ Department of Radiation Oncology, Juravinski Cancer Center, Hamilton, Ontario, Canada

**Keywords:** AMPK, ionizing radiation, stress, cell cycle, ATM

## Abstract

Lung cancer is the most fatal malignancy worldwide, in part, due to high resistance to cytotoxic therapy. There is need for effective chemo-radio-sensitizers in lung cancer. In recent years, we began to understand the modulation of metabolism in cancer and its importance in tumor progression and survival after cytotoxic therapy. The activity of biosynthetic pathways, driven by the Growth Factor Receptor/Ras/PI3k/Akt/mTOR pathway, is balanced by the energy stress sensor pathway of LKB1/AMPK/p53. AMPK responds both to metabolic and genotoxic stress. Metformin, a well-tolerated anti-diabetic agent, which blocks mitochondria oxidative phosphorylation complex I, became the poster child agent to elicit AMPK activity and tumor suppression. Metformin sensitizes NSCLC models to chemotherapy and radiation. Here, we discuss the rationale for targeting metabolism, the evidence supporting metformin as an anti-tumor agent and adjunct to cytotoxic therapy in NSCLC and we review retrospective evidence and on-going clinical trials addressing this concept.

## BACKGROUND

### Non-small cell lung cancer - standard therapy and outcomes

Lung cancer is the leading cause of cancer mortality worldwide accounting for 24% of global cancer related deaths. Each year, in North America over 250,000 patients are diagnosed with lung cancer and 180,000 die from the disease (American and Canadian Cancer Societies, 2016) [1]. Non-Small Cell Lung Cancer (NSCLC) represents 80% of all cases and includes three main histologies, adenocarcinomas, squamous cell carcinomas and large cell carcinomas. Surgery [2, 3] and modern stereotactic body radiotherapy (RT) (SBRT) [4] provide reasonable control for early stage tumors. However, locally advanced NSCLC is frequently in-operable and treated with combined Chemo-RT [5], with poor outcomes [6]. Current standard chest RT for locally advanced NSCLC (60-63Gy in 30 fractions) was developed more than 30 years ago [7] and is associated with 60-85% rates of recurrence [8]. Platinum-based concurrent Chemo-RT, established in late 1990s as standard therapy for locally advanced NSCLC [9, 10], shows a median survival of 24-28 months in studies with modern radiotherapy [11]. Unfortunately, escalation of RT dose (74Gy) failed to show benefit in phase III studies (RTOG-0617) [11]. There is an obvious need for well-tolerated therapies to enhance the response of NSCLC to Chemo-RT.

### Molecular pathways of cancer cell survival and radio-resistance

**Figure 1:**
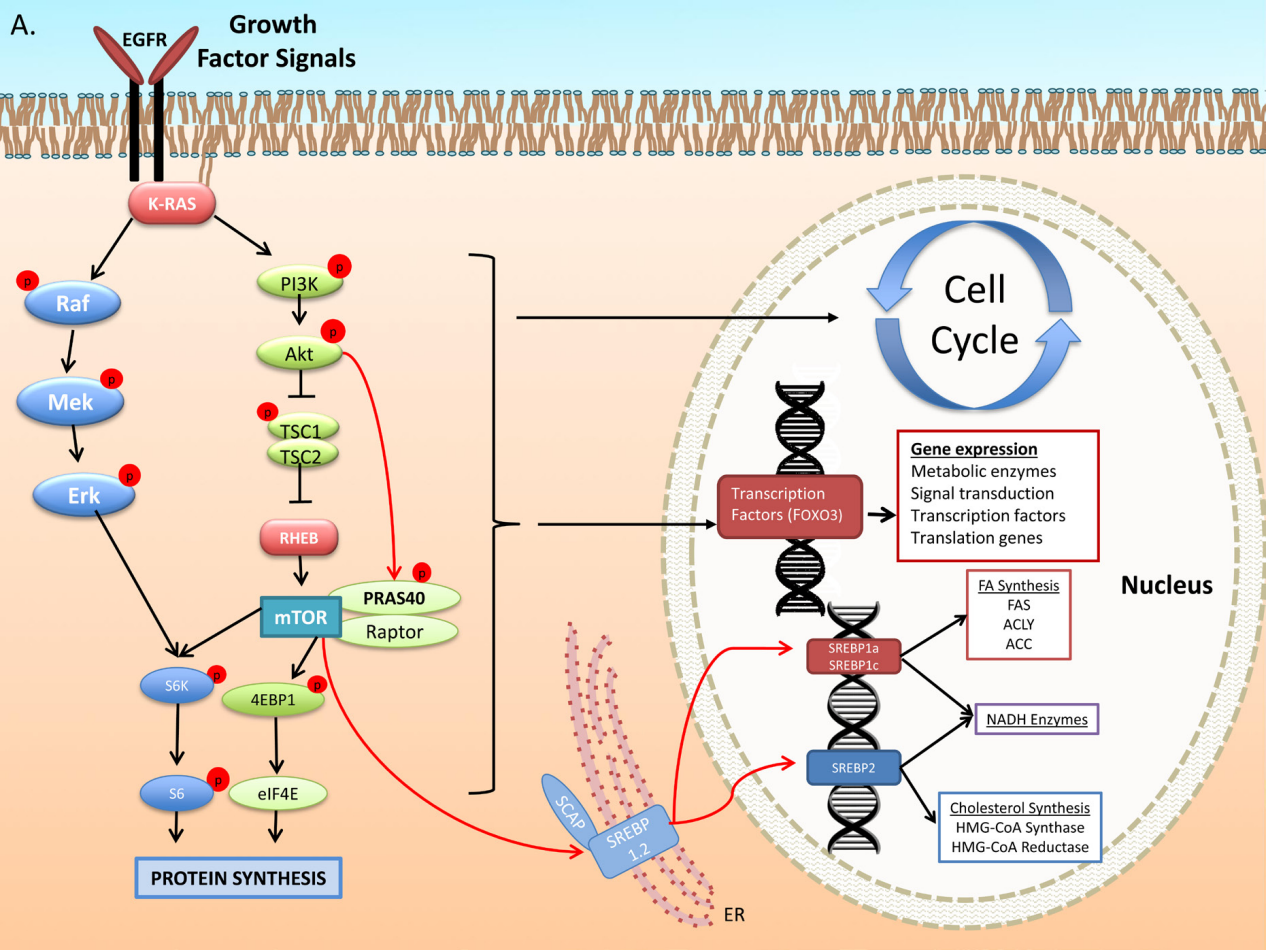

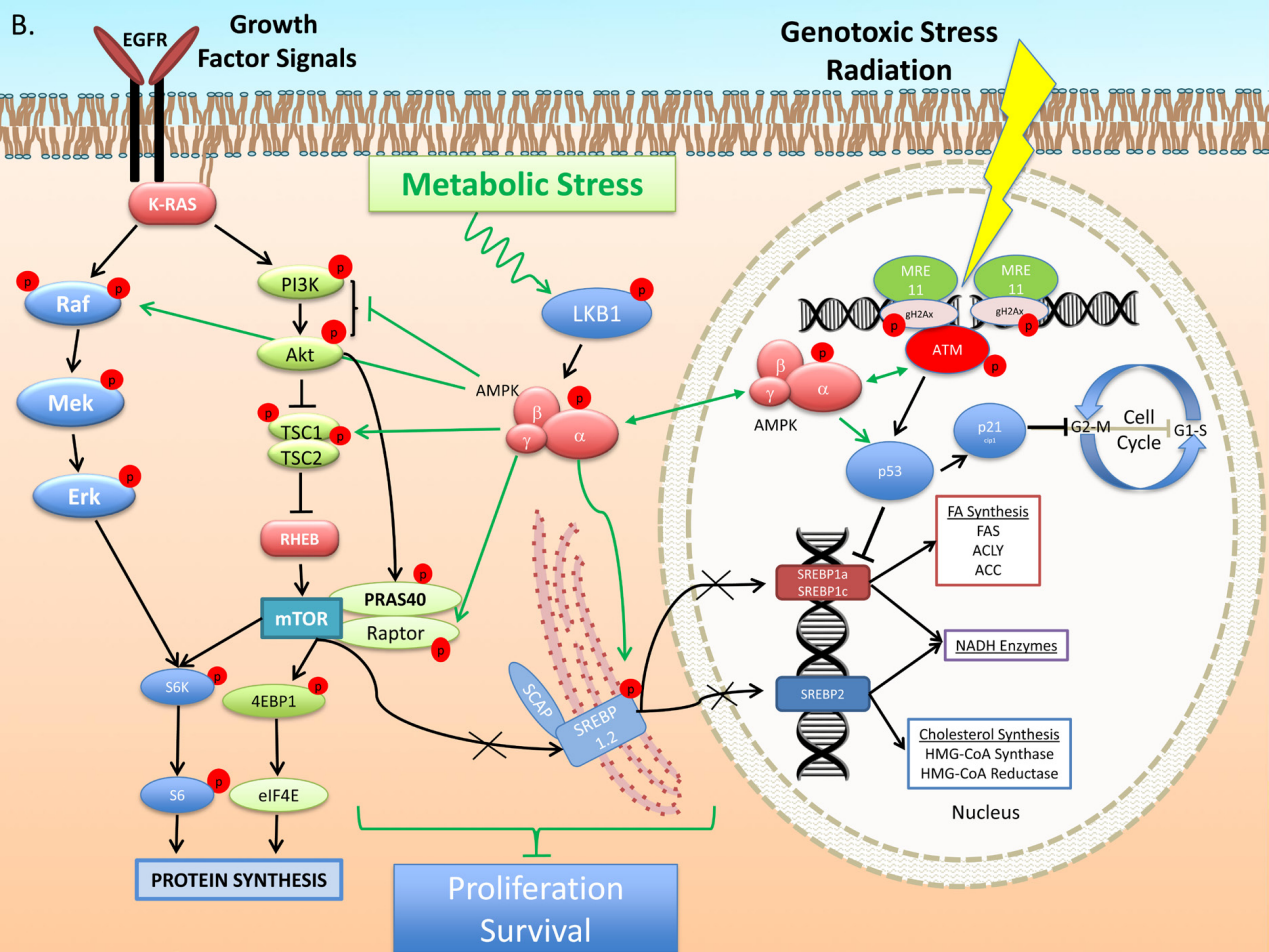
Growth Factor and DNA Damage Response (DDR) activated signal transduction: regulation of cell cycle, and metabolic gene expression **A.** The Epidermal Growth Factor Receptor (EGFR) activates signaling pathways that mediate, gene expression, protein and lipid synthesis, growth of cellular biomass and survival after cytotoxic therapy [146]. EGFR transduces signals through the well-described kinase pathways initiated by Ras, including the Raf/Mek/Erk and Phosphatidyl-inositol 3-kinase (PI3k)/Akt/mammalian Target of Rapamycin (mTOR). Akt activates mTOR through, i) phosphorylation and inhibition of Tuberous Sclerosis Complex 2 (TSC2), which inactivates the mTOR activating GTP-binding protein Rheb and/or ii) phosphorylation of PRAS40 a member of mTOR complex 1 (mTORC1) (one of the two functional complexes of mTOR that includes mLST8/Gbl and the scaffold protein Raptor [147]). mTORC1 promotes Cap-dependent gene expression and translation through phosphorylation-mediated activation of p70^S6^-kinase (p70^s6k^) and phosphorylation-mediated inhibition of translation initiation inhibitor eIF4E binding protein 1 (4EBP1) [146, 147]. The PI3k-mTOR pathway stimulates glucose uptake and de novo lipogenesis (DNL). Regulation of the latter is mediated at the transcriptional, translational and post-translational level. PI3k-Akt facilitate cleavage of Sterol Regulatory Element-Binding Proteins (SREBP) 1a, c and 2 [148] which, translocate to nucleus to stimulate expression of lipogenic enzymes mediating both fatty acid and cholesterol synthesis. Transcription of ACLY, ACC1 and FASN is stimulated by the PI3k-Akt pathway through SREBPs [149, 150]. SREBP1a and 1c regulate genes involved in FA metabolism while SREBP2 regulates cholesterol synthesis genes [150]. B. Metabolic stress activates a metabolic stress response signals through mediators, such as Liver Kinase B 1 (LKB1), which phosphorylates and activates AMP-activated kinase (AMPK). AMPK suppresses mTOR activation through dual action to phosphorylate and enhance the activity of Tuberous Sclerosis Complex 1 and 2 (an inhibitor of mTOR) and through inhibitory phosphorylation of mTORC1 protein Raptor. Further, it suppresses lipogenic gene transcription through inhibitory SREBP phosphorylation that prevents cleavage and nuclear translocation to induce transcription. Genotoxic Stress induced by ionizing radiation activates the DNA Damage Response (DDR). Chromosomal damage is recognized by MRE11 complex and ATM, a key mediator of DDR, which phosphorylates histone H2Ax (γH2Ax), a step leading to recruitment of molecular DNA repair complexes at the sites of strand breaks. ATM induces p53 expression and phosphorylation and expression of the cyclin dependent kinase inhibitor p21^cip1^, which facilitates the G1-S and G2-M checkpoints. Further, p53 blocks lipogenic gene expression at the transcriptional level. In lung cancer cells ATM induction of p53-p21^cip1^ appears to be mediated through AMPK [89]. Genotoxic stress, such as radiation, induces in lung cancer cells and tumors sustained activation of the ATM-AMPK-p53-p21^cip1^ and suppression of the Akt-mTOR pathways [73, 89, 151].

The Epidermal Growth Factor Receptor (EGFR), the oncogene K-Ras, the tumor suppressors TP53 and Liver Kinase B 1 (LKB1) are the most frequently mutated genes in NSCLC [12]. EGFR is an established driver of growth and survival (Figure 1A). It is mutated in 7-19% of adenocarcinomas and over-expressed in up to 50% of lung cancers [12]. EGFR inhibitors have been investigated intensively in the last 15 years [13]. Pre-clinical studies showed radio-sensitization of NSCLC to such inhibitors [14], and phase II studies showed promising results (IDEAL 1-2). However, phase III trials (INTACT-1/2) did not show improved response to chemotherapy [13]. Although the chimerized monoclonal antibody against EGFR cetuximab showed promising results in a phase II study (RTOG 0324) [15], it did not demonstrate benefit with either standard (60Gy) or dose-escalated (74Gy) Chemo-RT in phase III trials (RTOG-0617) [6].

The oncogene Ras is activated by EGFR to attract and activate multiple effector pathways promoting growth, survival and resistance to cytotoxic therapy (Figure 1A). Of the three mammalian Ras genes (H, K and N), K-Ras has the highest frequency of mutations in NSCLC (6.5% in squamous cell and 26% of adenocarcinomas, in Western patients) [12]. Lung adenocarcinomas associated with smoking reveal mutations in codons 12 or 13 of this gene, involving typically G-T transitions. K-Ras activates Phosphatidylinositol 3-kinase (PI3k) and Akt, which regulate the mammalian Target of Rapamycin (mTOR) complex 1 (mTORC1) [16] (see Figure 1A). Preclinical studies with PI3k and mTOR inhibitors did show sensitization of NSCLC xenografts to radiation [17, 18]. Currently, there is no evidence of clinical benefit of such inhibitors in combination with RT or standard Chemo-RT in lung cancer. mTOR inhibitors are reported to have a significant risk for pneumonitis (relative risk of 31 for mild- and 8.8 for grade 3-4) [19], which questions their use in lung cancer, particularly in combination with RT. These agents are suspected to have failed to show benefit due to a feedback activation of Akt, leading to resistance to cancer therapies [20].

### Radiotherapy: DNA damage-activated signaling pathways

RT kills cancer cells through lethal DNA double strand breaks (DSB). Sub-lethal doses permit DNA repair and survival initiated through detection of DNA breaks by the MRE11 complex and the kinase Ataxia Telengiectasia Mutated (ATM) [21–23] (Figure 1B). ATM and ATR (ATM and Rad3-related) regulate genotoxic stress-induced cell cycle checkpoints to facilitate DNA repair and preserve genomic stability [24], through phosphorylation and activation of p53 [25] and checkpoint kinases (Chk1 and Chk2) [26]. p53 and cyclin-dependent kinase (CDK) inhibitor p21^cip1^ mediate G1-S and G2-M checkpoints through, Cdk2-cyclin E [27] and Cdk1-cyclin B [28], respectively.

## CANCER METABOLISM

Hypoxia of the tumor microenvironment stimulates anaerobic metabolism in tumors, but even in the presence of oxygen tumors maintain anaerobic glycolysis, a paradox that Otto Warburg described nine decades ago [29, 30]. Today, studies continue to clarify the degree of metabolic adaptation taking place in cancer cells to meet the demands of rapid proliferation. Cancer cells exhibit enhanced glucose, protein and lipid metabolism, stimulated by the EGFR-PI3k-Akt-mTOR pathway [30]. These events lead to the Warburg phenotype to support unrestrained cell division. The enhanced metabolic activity of tumors contributes to cancer diagnosis and staging. For example, the high levels of glucose uptake in solid tumors forms the basis of positron emission tomography (PET), using ^18^F-labeled 2-deoxy-D-glucose (F^18^-FDG) as a marker of glucose uptake and tumor metabolic activity.

### Glucose transport and glycolysis

**Figure 2:**
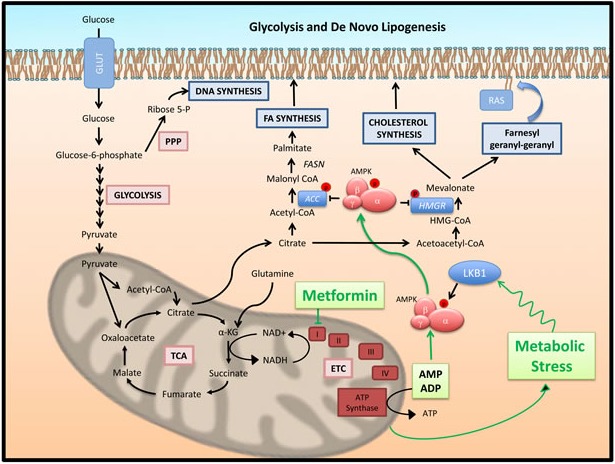
Glucose metabolism and lipogenesis, post-translational regulation by AMPK: Mechanism of action of metformin Glucose provides cancer cells with substrates not only for energy production but also for biosynthesis of proteins, nucleic acids and fats. Anaerobic glycolysis generates, (i) ribose 5-phosphate to support nucleotide biosynthesis through the pentose phosphate pathway and (ii) pyruvate to generate Acetyl CoA to feed the Krebs (TCA) cycle. The TCA cycle supports the electron transport chain (ETC) to produce ATP from AMP and ADP. However, TCA produced citrate is also transported to the cytoplasm by the tricarboxylate transporter to be cleaved by ATP Citrate Lyase (ACLY) and generate cytoplasmic acetyl-CoA. This is a crucial common step for the initiation of fatty acid (FA) and cholesterol synthesis. A rate limiting step in FA synthesis is the generation of malonyl-CoA from acetyl-CoA, catalyzed by Acetyl-CoA Carboxylase (ACC). Malonyl-CoA is further converted to palmitic acid by fatty acid synthase (FASN) [36]. On the other hand, conversion of acetyl-CoA to acetoacetyl-CoA, HMG-CoA and finally mevalonate initiates the pathway of synthesis of cholesterol and isoprenoids. A rate limiting step of this pathway is the conversion of HMG-CoA to mevalonate catalyzed by HMG-CoA reductase (HMGR). Products of the mevalonate pathway are important for tumor cell growth. Cholesterol is required for structure and function of plasma membranes, while isoprenoids (farnesyl-PP and geranyl-PP diphosphates) mediate the post-translational modification of oncogenes such as Ras [38]. Metformin mediates in cells a state of mild metabolic stress through inhibition of aerobic energy production at the mitochondria. It blocks the oxidative phosphorylation (OxPhos) complex I leading to enhancement of cellular AMP/ADP levels and stimulation of AMPK activity through binding to the enzyme's γ-subunit. The metabolic stress induced by metformin also induces LKB1 induced AMPK phosphorylation on AMPKα subunit T172 leading to greater activation. Through AMPK metformin blocks energy consuming biosynthetic pathways such as DNL. At the post-translational level, activated AMPK phosphorylates and inhibits both ACC and HMGR, leading to blockade of both fatty acid and cholesterol synthesis. Metformin blocks lipogenesis and this may be one of the key actions mediating its cytostatic activity.

Epithelial tumor cells express increased number of plasma membrane facilitative glucose transporters (GLUTs) [31] and glycolytic enzymes to mediate enhanced glucose uptake and catabolism (Figure 2). Loss of p53 and activation of the Akt pathway is critical in promoting the glycolytic phenotype of cancer cells by stimulating, i) transcription and translation of GLUT1 glucose transporter in cancer models [32, 33] and ii) hexokinase activity and localization with the mitochondria. This localization commits glucose to glycolysis once it is phosphorylated by hexokinase to glucose-6-phosphate, and is involved in Akt-mediated prevention of early apoptotic events [34].

### *De novo* lipogenesis (DNL)

Tumor cells activate endogenous lipid bio-synthesis to maintain fatty acid and cholesterol levels required for elevated rates of cellular replication. Early cancer research suggested that this takes place despite adequate extracellular lipid supply [35, 36]. Energy storage is perhaps the least important function of DNL in cancer since this function supports, i) the supply of building blocks for membrane biogenesis, ii) second messenger function, and iii) post-translational modification of signaling proteins, all of which are vital for rapidly dividing cells. Catabolism of glucose (glycolysis), lipids (mitochondrial β-oxidation) and proteins (glutamine) converge at the Krebs cycle to facilitate not only the generation of energy through oxidative phosphorylation (OxPhos), but also the generation of citrate to support lipogenesis [37]. Citrate is converted to acetyl-CoA by ATP Citrate Lyase (ACLY) (Figure 2), which supports both fatty acid and cholesterol synthesis. Acetyl-CoA Carboxylase (ACC) and Fatty acid synthase (FASN) are rate limiting steps in fatty acid synthesis, while HMG-CoA Reductase (HMGR) has this role for the mevalonate - cholesterol synthesis pathway and is the target of the anti-cholesterol agents statins [38].

The significance of DNL in cancer is evident by the up-regulation of lipogenic genes in many aggressive tumors including lung, breast, prostate and colon cancer [39, 40]. Increased expression and activation of ACLY is a negative prognostic factor in lung and colon cancer, and is associated with poor survival [41, 42]. Molecular or biochemical blockade of ACLY suppresses DNL and inhibits NSCLC cell growth [41, 43, 44], and combined inhibition of ACLY and HMGR by lovastatin enhanced the anti-proliferative effects of ACLY inhibition. Interestingly, combining ACLY knockdown and statin treatment also blocked PI3k/Akt and Mitogen Activated Protein Kinase (MAPK) signaling in EGFR and K-Ras mutant NSCLC lines [45]. Biochemical inhibition of ACC was also found to be toxic for NSCLC cells [46, 47]. Recently, Svensson et al (2016) [47] showed inhibition of ACC affected NSCLC cell and tumor growth, enhanced cytotoxic effects of chemotherapy, and observed increased activity in K-Ras mutant p53^−/−^ and K-Ras mutant - LKB1^−/−^ mouse models. While FASN inhibition has been shown to inhibit growth of other cancer types [48, 49], no significant work has been done in NSCLC. There is strong evidence that growth factor receptor pathways mediate expression of FASN through PI3k and Akt [36]. This suggests that DNL is potential target for mitigating NSCLC growth. However, development of compounds that could target DNL pathways and simultaneously activate AMPK and p53, could have advantages in slowing malignant growth by targeting cell cycle progression and enhancing radiosensitivity.

### LKB1 - AMPK pathway

The anabolic events at the cancer cell plasma membrane, cytoplasm and mitochondria are also under the tight regulation of the Liver Kinase 1 (LKB1) - AMP-activated (AMP-activated kinase) kinase pathway. LKB1 is a tumor suppressor that is mutated in Peutz-Jeghers syndrome and is associated with hamartomas, primary gut polyps, breast, colon, and lung cancer [50]. Studies show up to 30% rate of *Lkb1* point mutations and deletions in NSCLC, ubiquitous expression of LKB1 in adult lung bronchial epithelium and a progressive loss of LKB1 as pre-malignant adenomatous hyperplasia progresses to frank invasive disease [50]. The highest rate of LKB1 defects is detected in lung adenocarcinomas (34%). LKB1 defects alone are early events in lung cancer carcinogenesis, but within K-Ras mutant tumors, they help develop an aggressive metastatic phenotype [51]. Low copy number of LKB1 gene was associated with higher risk for brain metastasis in patients with advanced NSCLC expressing mutant K-Ras [52].

LKB1 mediates many of its metabolic and anti-proliferative functions through phosphorylation and activation of the AMPK. This is an evolutionarily-conserved sensor of metabolic stress that at times of low energy levels, i) inhibits anabolic processes including energy storage, cellular growth and proliferation, and ii) increases nutrient uptake and catabolism to enhance energy availability [50]. Homologues of AMPK in yeast and *C. Elegans* were shown to regulate fundamental processes such as cellular growth, survival under environmental stress and longevity [53]. Human AMPK is a heterotrimer, consisting of a catalytic α-subunit, and regulatory β and γ subunits. In humans two α (α1 and α2), two β (β1 and β2) and three γ subunit (γ1, γ2 and γ3) isoforms exist [54]. AMPK is activated by direct LKB1 phosphorylation on the α-subunit at threonine 172 (T172), and by increases in the AMP/ATP ratio induced by natural metabolic stressors such as exercise, starvation, and hypoxia. At times of energy stress increased levels of AMP/ADP bind to four tandem cystathionine β synthase (CBS) repeats of the γ-subunit (now known as Bateman domains [55]) and induce an allosteric activation of AMPK leading to a 5-fold increase of the α-subunit catalytic activity [56]. However, phosphorylation of T172 within an activation loop of α-subunit, by LKB1, removes its auto-inhibition and stimulates the activity of the kinase domains by 100-fold [57]. The regulatory β-subunit of AMPK acts as a scaffold on which the α and γ-subunits can bind and form a functional AMPK heterotrimeric complex [56]. The β-subunit contains a carbohydrate (glycogen) binding domain (CBM), the role of which is still being elucidated. It likely aids in the localization of AMPK close to substrates that also bind glycogen, such as glycogen synthase [58]. The β1-subunit, specifically, undergoes post-translational modification by myristoylation and phosphorylation, which is required for AMPK localization and activity [59]. Phosphorylation of β1 subunit Ser108, in the CBM domain, leads to allosteric activation of the kinase which prevents the de-phosphorylation of T172. This takes place in response to direct AMPK β1 activators A-769662 and salicylate [60].

### AMPK regulation of signaling and metabolic events

#### Early events in growth signal transduction

AMPK is described to regulate both the PI3k-Akt-mTOR and the Raf-Mek-Erk pathway that stimulate gene expression, cellular growth and survival (Figure 1B). It is suggested to inhibit early steps of insulin and Insulin-like growth factor I (IGF-1) receptor signal transduction through an inhibitory phosphorylation of Ser^789^ on Insulin Receptor Substrate (IRS)-1 leading to reduced activation of PI3k-Akt axis [61], an event observed in many tumor types. Further, AMPK attenuates the B-RAF-MEK-ERK pathway, through phosphorylation of BRAF on Ser^729^. This event promotes its association with the adaptor 14-3-3 and disrupts its interaction with the Kinase Suppressor of Ras (KSR) 1, which mediates activation of the B-RAF-MEK-ERK pathway [62].

#### Protein synthesis

AMPK blocks protein synthesis through inhibition of mTOR complexes, mTOR complex 1 (mTORC1), and its downstream action (Figure 1A). This is mediated through, i) phosphorylation of the tumor suppressor Tuberous Sclerosis 2 (TSC2, on Thr1227 and Ser1345 residues), which activates the TSC1:TSC2 complex, and induces the GTPase activity (inhibits) the small G-protein Rheb, an activator of mTORC1 and ii) through phosphorylation and inhibition of the mTORC1 complex protein Raptor, preventing mTORC1 from phosphorylating downstream targets [63]. These events block regulation of mTORC1 targets such as the ribosomal p70-S6 kinase (p70^S6K^), that stimulates ribosomal activity, and the eukaryotic initiation factor 4E (eIF4E) binding protein 1 (4EBP1), which normally blocks initiation of translation. [64]

#### Lipogenesis

AMPK inhibits DNL through modulation of lipogenic gene expression as well as direct regulation of enzymatic activity. It inhibits transcriptional activity of sterol regulatory binding proteins (SREBP) 1c and 2 through phosphorylation on Ser372/374 [65] (Figure 1), a step that blocks proteolytic cleavage of SREBP and their ability to translocate to nucleus to activate lipogenic gene transcription. In this fashion, AMPK exerts global control over *de novo* fatty acid synthesis. However, AMPK is a key regulator of ACC that controls the synthesis of FA [66]. It inhibits ACC through phosphorylation of Ser79 of ACC1 and Ser212 on ACC2. G. Steinberg and colleagues (2013) examined the regulation of lipid homeostasis by ACC by inhibiting the ability of ACC to be regulated by AMPK. Generation of ACC1 and ACC2 knock-in animals, as well as double knock-in (DKI) ACC1/ACC2 animals, which carry substitutions of Ser 79 and 212, in the two genes with alanine [67], showed that such animals have elevated lipogenesis, lower fatty acid oxidation, insulin resistance, glucose intolerance and fatty livers [67]. ACC DKI animals become obese on high-fat diet and in that setting are refractory to the insulin-sensitizing and lipid lowering effects of metformin [67].

#### Cell cycle and apoptosis

A key effect of AMPK on proliferation is the mediation of a metabolic checkpoint and induction of apoptosis and autophagy [68]. AMPK blocks cell cycle by increasing total and phosphorylated levels of p53 and its downstream effector p21^cip1^. Jones et al (2005) [69] suggested in cancer cells that glucose deprivation stimulates AMPK that in-turn phosphorylates p53 on Ser15, leading to increase in p53 activity [70] and stabilization of the molecule [71]. Further, AMPK phosphorylates the CDK inhibitor (CDKI) p27^kip1^ on Thr198 to sequester it in the cytoplasm and promote survival in response to nutrient or growth-factor withdrawal [72]. Interestingly, we [73] and others [74, 75] described nuclear localization of AMPK. The potential role of AMPK in mitosis was discussed earlier (see Sanli et al (2014) [76]). Other mechanisms described to link AMPK and apoptosis include the inhibition of FASN and stimulation of the mitochondrial apoptotic pathway [77–79].

#### Transcription factors

Transcription factors, their co-activators, and histones are modulated downstream of AMPK to regulate gene expression and nuclear events leading to metabolic reprogramming and cell survival. AMPK is described to regulate transcription factors involved in hepatic gluconeogenesis, helping to regulate hepatic glucose output. In the context of cancer and NSCLC, several other transcription factors are affected by AMPK activation, including SREBP, p53, hypoxia induced factor 1α (HIF1α) (discussed below), and forkhead box 03a (FOXO3a). SREBP1c regulates fatty acid synthesis genes, and is inhibited by AMPK [80]. p53 which regulates expression of apoptotic proteins, including members of the Bcl-2 family and caspase 6, as described, is induced and stabilized by AMPK [81]. AMPK suppresses protein levels of HIF1α, which when induced by hypoxia, facilitates expression of several pro-survival genes, including vascular endothelial growth factor (VEGF), and glucose transporters (GLUT1) [82]. Finally, AMPK regulates forkhead box protein O3 (FOXO3a) by direct phosphorylation, which regulates glucose metabolism and apoptosis, during metabolic stress [83].

#### Sensor of genotoxic stress

AMPK is activated in response to both chemotherapy and RT. We demonstrated that therapeutic doses of RT (2-8Gy) induce potent time- and dose-dependent activation of AMPK in cancer cells [70]. Phosphorylated AMPK is detected in the nucleus followed by translocation of the activated complex to the cytoplasm. Interestingly, RT-induced activation of AMPK was not dependent on LKB1, as it was observed in LKB1-null A549 cells. This action of AMPK was blocked upon inhibition of ATM. We proposed that AMPK, i) transduces signals through a DNA damage response (DDR)-activated ATM-AMPK-p53-p21^cip1^ axis, ii) facilitates the DDR-induced G2-M checkpoint and iii) mediates RT-induced cytotoxicity in NSCLC [70, 76] (see models Figure 1B). AMPK subunit gene expression is acutely stimulated by RT in cancer cells [73]. Work with embryonic fibroblasts from genetically engineered mice (MEFs) lacking AMPK α-subunit expression, suggested that AMPK plays in stabilizing the basal activity of DDR and survival signaling pathways. We observed that lack of AMPK destabilizes both the ATM-p53 and the Akt-mTOR signals. Untreated AMPKα1,2^−/−^MEFs have enhanced ATM and p53 as well as Akt-mTOR signals but these pathways failed to respond to radiation. These cells lacked a G2-M checkpoint response and showed evidence of radio-resistance [73]. In human NSCLC xenograft models RT leads to a chronic sustained expression and activation of the entire ATM-AMPK-p53/p21^cip1^ pathway but inhibition of mTOR signals [84] that is associated with inhibition of tumor growth, expression of apoptosis markers and inhibition of angiogenesis [84]. RT-induced expression and activation of AMPK is mediated through sestrin 2, a member of a family of stress-induced genes that is activated by RT and mediates radio-sensitization of breast and NSCLC cells [85]. Overall, RT causes sustained activation and increased expression of AMPK and its effectors in lung cancer cells and tumors leading to inhibition of survival.

### Targeting cellular metabolisms to enhance response to cytotoxic therapy

AMPK's ability to regulate cellular growth and cell cycle revived the concept that targeting cellular metabolism may be able to control tumor growth. This triggered investigation of agents mediating metabolic stress in cells but the anti-diabetic agent metformin gained the greatest popularity due to its favorable toxicity profile, wide-spread use, low cost and excellent tolerability in non-diabetics also.

## METFORMIN

Metformin (1-(diaminomethylidene)-3,3-dimethyl-guanidine) is a biguanide, a class of anti-diabetic drugs containing two linked guanidine rings that was derived from galegine, a guanidine found in French lilac (galega officinalis). Two other biguanides, phenoformin and buformin have potent activity and were used earlier but were withdrawn from clinical use due to increased risk of lactic acidosis. Metformin is an effective and well-tolerated anti-diabetic agent used by more than 120 million patients worldwide [86] and it is also indicated for the treatment of polycystic ovary syndrome and non-alcoholic liver disease.

### Metformin and cancer

#### Pre-clinical studies - molecular mechanism of action

**Figure 3:**
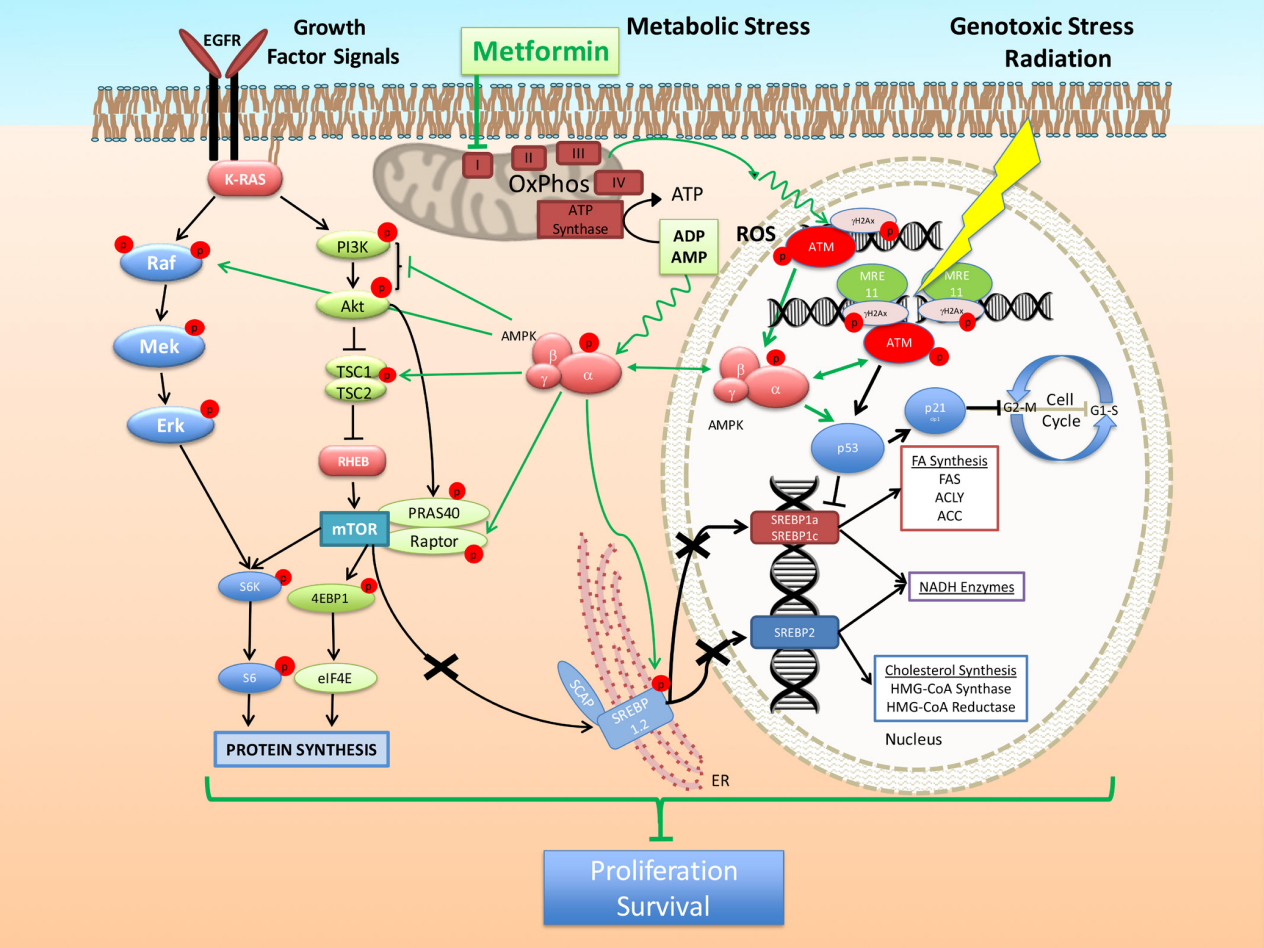
Model of the mechanism of action of metformin alone and in combination with cytotoxic therapy Inhibition of mitochondria OxPhos complex I by metformin creates in cancer cells a metabolic stress characterized by increased levels of AMP/ADP and potential generation of Reactive Oxygen Species (ROS). The first leads to AMPK activation through binding on the AMPK γ subunit, while the second is likely responsible for the observed trigger of DDR, activation of ATM and induction of γH2Ax foci and activation of AMPK. These effects enhance further the activation of AMPK mediated by genotoxic stress leading to improved suppression of the downstream events such as the mTOR pathway, metabolic gene expression, cell growth, cell cycle progression and survival.

Metformin inhibits complex I of the mitochondrial OxPhos chain, leading to increased levels of ADP and AMP and activation of AMPK [56] (Figures 2, 3). Thereby, metformin induces all pathways, metabolic and cytostatic events attributed to AMPK [87]. Many studies observed anti-tumor activity of metformin in NSCLC cell lines but fewer examined the drug in combination with targeted or cytotoxic therapy (see Table 1). Although, most groups analyzed very similar or the same NSCLC cell lines, significant controversies exist in the reported mechanism of action of metformin and phenformin. Studies observed anti-tumor activity of metformin in most lung cancer lines and histologies, including small cell lung cancer, but responses were generally detected at high (mM) concentrations [88, 89].

**Table 1:** Pre-clinical studies of metformin in lung cancer: Investigation of metformin alone and in combination with chemotherapy and radiation

Treatment Type	Cell /Animal Model	Metformin Dose	Combination Treatment	Effect of Treatment/ Mechanism of action	Author
**None**	Lewis Lung LLC1 (LKB1^+ vs shRNA^)	5 mM 50mg/kg/day	---	Anti-proliferative activity: metformin blocked high-energy diet-induced tumor growth, ***LKB1 loss enhances response in low glucose media, LK81-independent activity in high glucose***	Algire et al (2008/2011) [142],[143]
	CALU-1, CALU-6	5-20 mM	---	K-Ras mutant cells, ***Caveolin-1-dependent activation of AMPK and growth suppression***	Salani et al (2012) [93]
	RERF-LC-Al, A549, IA- 5, Wa-hT	0.5-4 mM	---	Inhibition of surviving fraction in all histologies	Ashinuma et al (2012) [88]
	A549, H460, A427, H838, H157 Adenoviral Cre- induced K-Ras^6120^- floxed LKB1 / p53	2-20mM *Studied mainly phenformin*	---	K-Ras mutant cells: metformin activated AMPK and inhibited mTOR but did not induce apoptosis; *lack of LKB1 but not p53 enhances tumors activity of Phenformin;* K-Ras^G120^ — LKB1^−/−^ but not K-Ras^G120^-p53-^/−^ cells sensitive to phenformin	Shackelford et al (2013) [92]
	A549, H1299	0.3-10 mM	---	***Metformin inhibits lung cancer through blockade of mitochondria metabolism** but not through LKI31 or AMPK*	Griss et al (2015) [95]
	H460, H1299	5-10mM	---	Inhibition of survival — induction of apoptosis: ***LKB1-independent, AMPK-dependent***	Guo etal *(2016) [94]*
	A549	250 mg/kg/day	---	Metformin inhibits mutant K-Ras (A549) but not wild type lines	Ma *et al* (2013) [91]
**Prevention**	NNK-induced lung tumorigenesis in A/J mice	1-5 mg/ml.	---	Suppressed tumorigenesis	Memmott et al (2010) [144]
**Radiation**	A549, H1299, SK-MES	5 μM - 5 mM	Ionizing Radiation	Inhibition of proliferation, clonogenic survival, pro-apoptotic and radio-sensitization: ***LK81-independent, ATM- AMPK-dependent***	Storozhuk et al (2012) [89]
**Chemotherapy Targeted therapy**	A549 grafted into (nu*/nu*) mice	200 μg/ml	Doxorubicin Carboplatin Paclitaxel	Suppressed tumor growth and prolonged remission	Iliopoulos et al (2011) [116]
	A549 cells and xenografts	0.01-100 mmol/L	Paclitaxel	Synergistic effect with chemotherapy	Rocha et al(2012) [115]
	A549	5-50mM	Cisplatin	Inhibition of survival, induction of apoptosis, chemo-sensitization	Wang etal (2015) [97]
	A549	5-20 mM	Cisplatin	Metformin induced *resistance to Cisplotin*	Ashinuma etal (2012) [88]
**Targeted therapy/ Chemotherapy**	EGFR mutant PC9 cells and xenografts	10 mM 150mg/kg/d IP	Gefitnib Cisplatin	no cisplatin chemo-sensitization with metformin, suppression of Gefitinib resistant tumor growth	Kitazono etal (2013) [113]
**Targeted therapy**	CALU-3, CALU-3 GEE-R, H1975, H1299, GLC82 cells H1299 and CALU-3 (EGFR resistant) xenografts	0.1-20mM 200 mg/ml. in drinking water	Gefitnib	Synergistic anti-tumor activity of metformin and gefitinib in-vitro and in-vivo, ***LKB1-dependent***	Morgillo etal (2013) [112]

#### Role of LKB1

In mouse Lewis lung model, Algire et al (2011) [90], detected opposite roles of LKB1 on the response of LLC1 cells to metformin depending on glucose availability. In cells grown in standard high glucose media (25mM), LKB1 knockdown blocked AMPK and ACC phosphorylation and abolished metformin-induced growth inhibition. However, in low glucose media (2.5mM) lack of LKB1 enhanced significantly the cellular sensitivity to metformin. These findings are contrary to the notion that the LKB1-AMPK axis mediates tumor suppression and indicate that LKB1 may trigger a specific mechanism of resistance to tumor suppressive effects of metabolic stress.

#### K-Ras

Adenocarcinoma cells harboring K-Ras with or without loss of LKB-1 expression are reported to show greater sensitivity to biguanides [88, 91]. However, the mechanism behind this sensitivity is not defined. One would expect that enhanced signaling downstream of K-Ras (including PI3k-Akt-mTOR) would stimulate a glycolytic and lipogenic phenotype that would resemble the Warburg effect and mediate independence from OxPhos, the site of action of biguanides. Shackelford et al (2013) [92], studied mutant K-Ras^G12S^-LKB1^−/−^ adenocarcinoma lines and detected LKB1-dependent activation of AMPK and blockade of mTOR by both metformin and phenformin when wild type LKB1 was transfected, but detected apoptosis only in response to phenformin. Using the adenovirus Cre-induced lung specific model of K-Ras^G12D^ expression they studied the impact of floxed alleles of p53 or LKB1. p53 loss is well-described to increase significantly tumor burden, metastasis and radio-resistance in this model but they found that loss of LKB1, and not p53, mediated phenformin sensitivity.

#### AMPK

A number of groups verified the dependence of metformin action on AMPK activity. In the background of K-Ras mutation, Salani et al (2012) [93] suggested that AMPK activation is dependent on Caveolin-1. We detected LKB1-independent but AMPK-dependent inhibition of survival of adeno- and squamous cell carcinoma lines with microM concentrations of metformin [89]. Similar results were obtained by Guo et al (20156) [94], albeit with higher drug doses. Inhibition of proliferation and clonogenic survival was independent of p53 or LKB1 status. Metformin was equally active in A549 (K-Ras^G12S^-LKB1 null) and in H1299 (N-Ras^Q12K^-p53 null) cells and xenografts [89], and in squamous SK-MES (p53 null) cells.

Recent studies, using the same cell lines (H1299, A549) suggested that the response to metfromin is mediated solely by, i) suppression of mitochondria metabolism, which suppresses vital cellular functions, like lipogenesis [95] or ii) by mitochondria-mediated apoptosis [96].

#### ATM

Metformin induces expression of ATM and activation, detected as ATM phosphorylation and induction of γH2Ax foci, and this is required for metformin activation of AMPK [89]. The mechanisms by which metformin activates ATM and how this contributes to AMPK activation are not well understood. Other groups also described the concept of ATM-induced AMPK activation is response to hormone (IGF-I) [97] and cytotoxic therapy (etoposide) [98], metabolic stress [99] and in response to metformin in esophageal and epidermoid cancer cells [100, 101]. The site of metformin-induced ATM activation may not be limited to the nucleus. ROS were suggested to activate ATM in the cytoplasm in breast cancer cells, leading to activation of LKB1-AMPK-mTOR axis [99]. Other groups also detected nuclear activation of ATM. Vazquez-Martin et al (2011) [101] observed presence of γH2Ax foci and ATM phosphorylation in the absence of p53 binding protein foci (indicative of DNA strand breaks) or positive comet assays. They suggested that metformin may mediate changes in the chromatin structure that triggers activation of DDR and phosphorylation of ATM, leading to checkpoint activation. ATM is felt to function as a guardian of the genome in the face of genotoxic stress induced by cytotoxic and metabolic insults [101, 102].

#### Mitochondria - ROS

Whether metformin induces or reduces ROS levels in cells remains controversial. It was suggested that blockade of complex I leads to reduction of mitochondrial ROS production [103], but OxPhos blockade leads to acidosis and others did show enhanced ROS levels with prolonged metformin incubation in breast cancer cells [104]. It is possible that blockade of mitochondrial OxPhos by metformin induces a slow generation of ROS, which diffuse into the nucleus to induce activation of ATM in the absence of true DNA breaks (see model of Figure 3). Although, the exact mechanism of ATM activation by metformin remains unclear, the presence of γH2Ax foci indicate that byproducts of metabolic stress indeed enter the nucleus and mediate a state of genomic stress that activates DDR. Alternatively, metformin may trigger cytoplasmic activation of ATM that subsequently shuttles into the nucleus. Future studies need to elucidate the exact mechanism by which ATM regulates AMPK.

#### Hypoxia induced factor (HIF)1α

HIF1α has a well-described role in supporting cellular survival in the hypoxic tumor micro-environment, a mechanism well-linked to radio-resistance and tumor metastatic potential [105, 106]. Although one would expect that induction of energy stress by metformin, through complex I inhibition, would likely induce HIF1α, studies demonstrate suppression of HIF1α by metformin [107, 108], which is suggested to contribute to the anti-tumor action of the drug [109]. In the absence of LKB1, HIF1α mediates expression of the Warburg metabolic phenotype and LKB1 and AMPK are shown to suppress transcription and translation of HIF1α, albeit by different mechanisms [110]. Whether the metformin-induced suppression of HIF1α involves LKB1 or AMPK or neither, this mechanism is dependent on the blockade of mitochondrial complex I. Wheaton et al (2014) [111] showed that expression of the metformin-resistant *Saccharomyces cerevisiae* NADH dehydrogenase NDI1 blocks the suppression of HIF1a by metformin, indicating the importance of OxPhos inhibition in suppressing HIF1α.

#### Interaction with cytostatic therapies

In our hands, the anti-proliferative effects of μM doses of metformin were comparable to those of EGFR (Gefitinib) and mTOR (rapamycin) inhibitors (supplemental data in Storozhuk et al (2013)) [90]. NSCLC cell line and xenograft work indicated that metformin could enhance the activity of EGFR inhibitor Gefitinib [112] and suppress tumor growth after Gefitinib withdrawal [113] (Table 1). Further, we showed that in NSCLC cells metformin enhances the anti-tumor activity of other AMPK-activating agents such as salicylate in both p53 null and LKB1 null cells, through a mechanism that involves AMPK β1 subunit and DNL [114] (Table 1).

### In combination with chemotherapy

Rocha et al (2011) [115] showed that in lung cancer models paclitaxel activates AMPK and inhibits mTOR, effects that are enhanced by metformin which enhances paclitaxel cytotoxicity (Table 1). While one group suggested that cisplatin may antagonize the anti-proliferative effects of metformin in some cell lines [88], other studies [116] suggested that metformin could function in combination with doxorubicin to reduce the chemotherapy dose needed to suppress tumor growth [116] and can mediate synergistic cytotoxicity with cisplatin [117, 118].

### Radio-sensitizing activity

We observed that metformin sensitizes lung cancer cells and tumors to RT [89] (Table 1). Metformin enhances the RT-induced activation of the ATM-AMPK-p53/p21^cip1^ axis, while it mediates suppression of the Akt-mTOR-4EBP1 pathway [89]. Importantly, we detected these effects of metformin at low micro-molar (5-100μM) doses. Metformin enhanced the induction of γH2Ax and total ATM levels in irradiated lung cancer cells and tumors, indicating a sustained increased expression and activation of ATM. This increase was present in metformin treated tumors even 8 weeks after RT [89]. Inhibition of ATM activity with knockdown using specific ATM siRNA or with the inhibitor KU60019 blocked both metformin- and RT-induced histone phosphorylation (γH2Ax) and AMPK activation. Further, knockdown of AMPK α-subunit, i) blocked metformin- and RT induction of p21^cip1^, ii) allowed activation of Akt and mTOR and iii) blocked the inhibition of lung cancer cell proliferation mediated by metformin and RT [89]. In NSCLC xenografts metformin given orally (in drinking water) at a dose of 250mg/kg/day enhanced the activation of ATM - AMPK - p53/p21cip1 axis induced by RT, inhibited tumor growth and enhanced the cytotoxicity of RT, inhibited angiogenesis and enhanced the induction of apoptotic markers. This work suggested that metabolic stress with metformin can be combined with RT to enhance the tumor suppressive effects of RT (Figure 3).

#### Metformin bio-availability in tumors

Some of the work described above, suggests that metformin could have direct activity in lung tumors at doses that can be achieved in human circulation. However, doubt developed in the scientific community, as to whether metformin is able to mediate direct anti-tumor activity and activate AMPK in tumor cells, since many studies showed activity of the drug in cancer cell lines only at mM concentrations of metformin that cannot be achieved clinically [87, 119, 120]. Diabetic patients are typically treated with 1-2.5g of metformin per day, a dose that achieves typically plasma levels of 7-10 μM [121, 122] but peak plasma concentrations of 30-40 μM have been described [122]. In our studies such plasma levels could mediate direct anti-tumor activity in lung cancer. The discrepancy between achievable doses of metformin in humans and the ones needed to mediate anti-tumor action in other tumors led groups to suggest that most of the anti-tumor activity of metformin may be due to the indirect hepatic action of the drug where, due to portal circulation, metformin reaches greater concentration [123]. Metformin action in liver could lead to improved control of glycemia and reduced circulating levels of insulin and IGF-1, which can reduced trophic effects on tumors. Nevertheless, recent studies examined metformin levels in the circulation of animals used in xenograft studies and in tumor tissue, using liquid chromatography and mass spectroscopy. Dowling et al (2016) [124] observed that intraperitoneal injections of 125mg/kg caused average plasma and tumor levels of metformin (of 145 μM and 77 μM, respectively), while delivery of the drug in the drinking water (at 5mg/ml) provided a stable average level of 34 μM and similar tumor concentration of 32 μM. At those concentrations of metformin caused activation of AMPK in colon carcinoma xenografts (HCT116), while activation of AMPK in the same cells *in vitro* required 10-20 mM metformin. In our studies with A549 and H1299 lung adenocarcinoma xenografts we observed sustained activation of AMPK with oral delivery of metformin in drinking water at a concentration of 250mg/kg/day (about 1.25mg/ml) [89]. It is suggested that, being a cation, metformin could accumulate in the mitochondria at 100-500 times higher concentrations due to the mitochondria membrane potential, leading to an effective inhibition of complex I. Overall, metformin should be able to mediate AMPK activation and anti-tumor activity, in human tumors with serum levels that are clinically achievable with standard anti-diabetic doses of the drug, indicating that metformin doses used in current clinical trials are appropriate.

## CLINICAL INVESTIGATION OF METFORMIN IN CANCER

### Retrospective clinical studies in cancer: Prevention vs improved response to cytotoxic therapy

Retrospective work has suggested that metformin may decrease the incidence of cancer in patients with type 2 diabetes [125, 126], and may be able to improve cancer treatment outcomes. Single center studies and a meta-analysis showed that metformin use was associated with a decrease in cancer mortality in all sites, including lung cancer (RR 0.66, *p* = 0.005) [127, 128]. Importantly, the beneficial effects of metformin were not shared with other hypoglycemic agents and potentially worse outcomes have been reported in patients treated with sulfonylureas and insulin [129–131].

#### Lung cancer prevention and outcomes

Reduced incidence of NSCLC was also reported with metformin in type II diabetic patient in some [128, 130, 132] but not all studies [133] (Table 2). A retrospective study from Cleveland Clinic observed that diabetic patients with lung cancer, who were previously exposed to metformin and/or another class of anti-diabetic drugs (thiazolidenediones: also known to activate AMPK), are less likely to present with metastatic disease, are more likely to present with an adenocarcinoma, and they may survive longer [132]. Tan and colleagues (2011) [134] suggested that locally advanced (LA-)NSCLC patients taking metformin had a median OS of 20 months compared to 13 months for those who took other hypoglycemic agents (insulin or other). This was also observed by retrospective analysis in other tumors such as esophageal, head and neck and prostate cancer [135–140].

**Table 2:** Retrospective clinical studies with metformin in lung cancer

Author	Number of subjects	Combination Treatment	Effect of Treatment
Incidence - Prevention			
Lai etal (2012) [1461	19, 624	---	Reduced risk of lung cancer by 39.45% in diabetic patients
Bodmer et al(2012) [1341	13, 043	---	Long-term use not associated with decrease risk of lung cancer in diabetic and non-diabetic patients
Noto etal (2012) [129]		---	Meta-analysis - significantly reduced risk of lung cancer in diabetic patients
Massone etal (2012) [133]	522		Lowered likelihood of developing cancer in diabetic patients
**Chemotherapy**			
Tan etal (2011)(135)	>4,000	First-line Chemotherapy	Improved PFS and OAS (median OAS 20 vs 13 months) in diabetic patients when compared to groups treated with insulin or metformin and insulin
**Chemo-Rediotherapy**			
Wink etal (2016)[141]	59 patients on metformin vs 632 controls	RT with concurrent cisplatin or cisplatin-etoposide chemotherapy	Significantly improved PFS and Distant metastasis free survival

### Retrospective results in LA-NSCLC treated with chemo-radiotherapy

Recently, Wink et al (2016) [141] suggested that metformin use is associated with improved Progression Free Survival (PFS) in patient with LA-SCLC that were treated with chemo-radiotherapy (Table 2). They reviewed patients treated in three radiation oncology departments in Netherlands, between 2008 and 2013, and identified 623 controls and 59 who received metformin at least during their radiotherapy course. Systemic therapy included either daily cisplatin or three-week cycle cisplatin-etoposide regimens, while chest RT was equivalent to 50 Gy or higher. Overall survival (OAS) and loco-regional recurrence free survival were not statistically significant between metformin users and non-users but PFS and distant metastasis free survival were found to be significantly increased in the metformin group (*p* < 0.01) [141].

**Figure 4:**
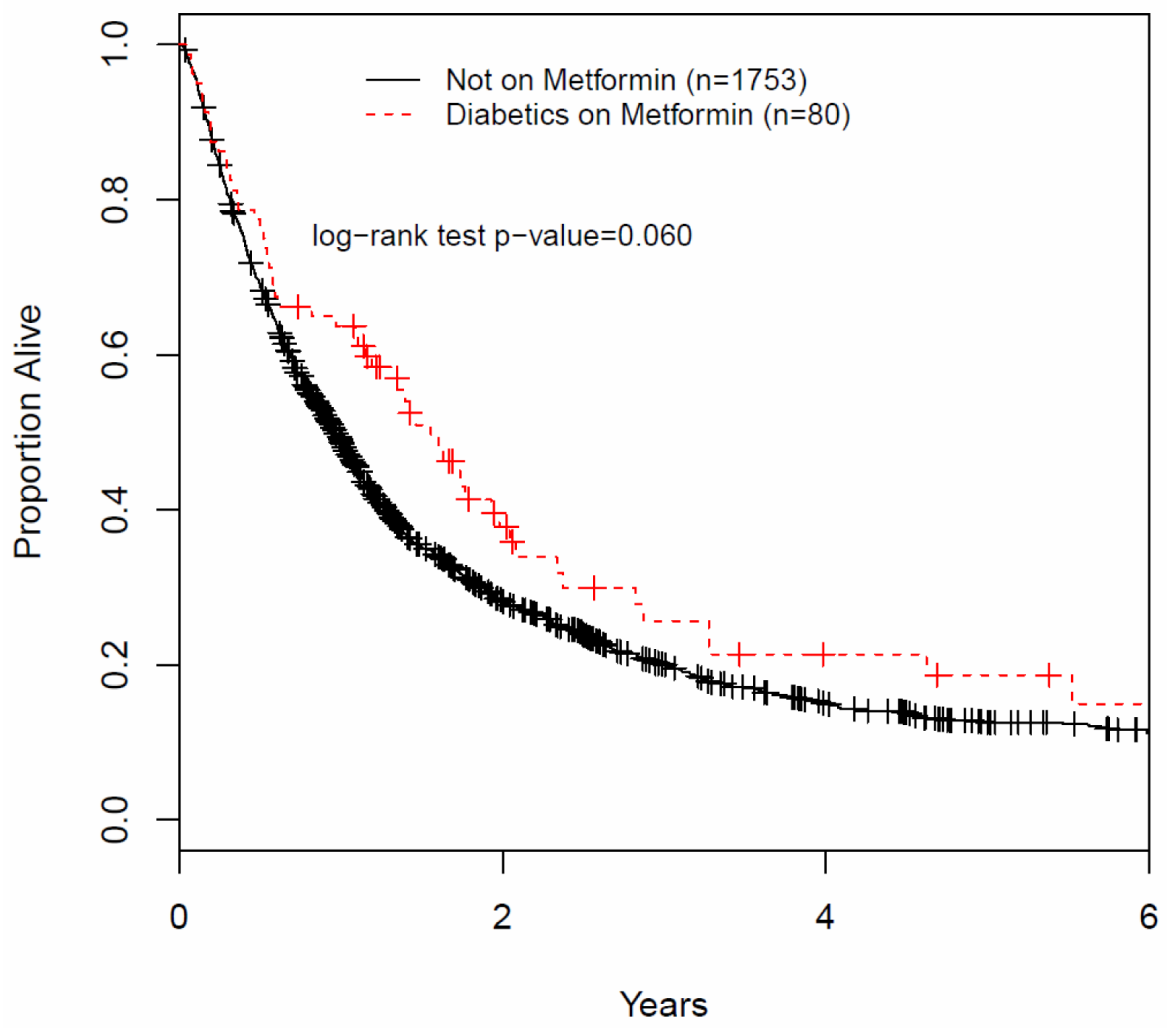
Overall survival of patients with LA-NSCLC treated with chemotherapy and radiotherapy, with or without metformin A retrospective case review of patients treated at the Juravinski Cancer Center, Hamilton, Ontario, in the period of 1998 - 2013. Patients with stage II and III disease were selected that received 50Gy or more of chest RT with or without concurrent chemotherapy. We identified 80 patients who were treated with metformin, as therapy for type II diabetes, during the period of cytotoxic therapy and beyond *vs* 1753 patient not treated with metformin. Patients received variable metformin doses of 1000 - 2500mg daily and the length of treatment with metformin after cytotoxic therapy was not determined. Patients and treatment characteristics of the two groups (patients NOT on Metformin vs ON Metformin) were similar: i) age: 68.82 +/− 10.72 and 68.79 +/− 8.38; ii) female to male ratio was 0.8 in both groups; iii) % of stage II: 14.3 *vs* 15.3; iv) % of stage III: 85.6 *vs* 84.6; v) % treated with chemotherapy: 77.05 *vs* 62.16, respectively.

We also pursued a retrospective analysis of patients with locally advanced NSCLC treated at the Juravinski Cancer Center (Hamilton, Ontario, Canada). Stage II-III patients were selected that received 50 Gy or more of chest RT with or without concurrent chemotherapy. Figure 4 illustrates a Kaplan-Meyer survival plot of the two groups. Median survival was 12.3 months for patients not treated with metformin *vs* 18.7 months for those who were treated with metformin. The benefit associated with metformin use appeared to diminish with prolonged survival. Results showed a trend for improved OAS in patients treated with metformin vs the controls, which approached but did not reach statistical significance (*p* = 0.06). These results are consistent with the observations of Wink et al (2016) [141] and other groups indicating that metformin may indeed be associated with improved outcomes in NSCLC. Recognizing the limited value of retrospective data, these and other results suggested the need to examine metformin in combination with standard cytotoxic therapy (Chemo-RT) in prospective controlled clinical trials.

### Active prospective cancer trials

Presently, there are over 130 clinical trials investigating metformin in cancer overall, while 12 studies focus on lung cancer (www.ClinicalTrials.govOctober 2016). Table 3 groups these studies in terms of their primary and additional intervention.

**Table 3:** Active clinical trials with metformin in lung cancer (0 www.ClinicalTrials.gov, Oct. 2016)

	NCT#	trial phase	Title	Institution	Investigation question	Status	Histology	stage	Other intervention	Comment
**2o Prevention**	NCT01717482	Phase II / Feasibility	Metformin as a Chemoprevention Agent in Non-small Cell Lung Cancer	Mayo Clinic	Feasibility of patient randomization to metformin as Chemo prevention	Active and Recruiting	NSCLC	IA-IIIA post-surgical reseaction	Observation	Status: post resection / metformin as chemoprevention
**Chemo-Radio-therapy**	NCT02109549	Observational	Influence of the Use of Metformin on the Overall Survival and Treatment-related Toxicity in Advanced Stage Non-small Cell Lung Cancer Patients.	Maastricht Radiation Oncology	Metformin use, insulin use, toxicity	Active NOT Recruiting	NSCLC	Locally Advanced NSCLC	Concurrent chemo radiation	Observing Non-diabetics vs diabetics not on metformin vs diabetics on metformin
	NCT02186847	Phase II Randomized	Chemotherapy and Radiation Therapy With or Without Metformin in Treating Patients With Stage III Non-small Cell Lung Cancer	NRG-LU001	metformin as sensitizer to chemo-radiotherapy	Active and Recruiting	NSCLC	IIIA-IIIB	Chemotherapy and Radiation	metformin delivered only concurrent with cytotoxic therapy
	NCT02115464	Phase II Randomized	Chemotherapy and Radiotherapy with or without Metformin for stage III locally advanced NSCLC (ALMERA)	OCOG-ALMERA	metformin as sensitizer to chemo-radiotherapy and as consolidation therapy	Active and Recruiting	NSCLC	IIIA-IIIB	Chemotherapy and Radiation	metformin delivered concurrent with cytotoxic therapy and as consolidation therapy × 12 months
**Radio-therapy alone**	NCT02285855	Phase II Randomized	Metformin in Non Small Cell Lung Cancer (NSCLC)	M.D. Anderson Cancer Center	metformin as radio-sensitiser to SBRT	Active and Recruiting		Stage I/II	Stereotactic body Radiotherapy (SBRT)	Non-surgical candidates due to Performance status
**Chemotherapy**	NCT02254512 / NCT02019979	Phase II Single arm	Metformin with a Carbohydrate Restricted Diet in Combination With Platinum Based Chemotherapy in Stage IIIB/IV Non-squamous Non-small Cell Lung Cancer (METRO)	Beth Israel Medical Center	Synergitic effect of metformin with platinum based chemotherapy	Active and Recruiting	Non-squamous Non-small-cell Lung	IIIB/IV	platinum based chemotherapy (carboplatin/ pemetrexed)	
**Chemotherapy / Targeted therapy**	NCT01997775	Phase II	Metformin in Stage IV Lung Adenocarcinoma	National Cheng-Kung University Hospital	Synergitic effect of metformin with chemo/targeted therapy with its role on lowering IL6	Unknown	Adeno-carcinoma of lung	IV	Chemotherapy or targeted therapy with Gefitinib	
	NCT01578551	Phase II Randomized	Metformin Plus Paclitaxel/Carboplatin/Bevacizumab in Patients With Adenocarcinoma. (NA_00052073)	Sidney Kimmel Comprehensive Cancer Center	Synergitic effect of metformin with chemo/monoclonal Ab therapy on survival, role of LKB1 gene status in tumors on response rate when drug added	Active and Recruiting	Adeno-carcinoma	Previously Untreated Advanced/Metastatic Pulmonary Adenocarcinoma	Paclitaxel/Carboplatin/Bevacizumab	T4NX disease (stage III B) with nodule(s) in ipsilateral lung lobe are not eligible measurable stage IV disease (includes M1a, M1b stages or recurrent disease
**Targeted Therapy**	NCT01864681	Phase II Randomized	Combination of Metformin With Gefitinib to Treat NSCLC (CGMT)	Daping Hospital and the Research Institute of Surgery of the Third Military Medical University	Synergitic effect of metformin with TKI	Active NOT Recruiting	NSCLC	Previously Untreated IIIB or IV	Gefitinib	Outcomes: PFS, OAS, IL6 levels
	NCT00659568	Phase I	Metformin and Temsirolimus in Treating Patients With Metastatic or Unresectable Solid Tumor or Lymphoma	London Health Sciences Centre	Synergitic effect of metformin with Tesirolimus(m-tor I)/established Max tolerance dose with tesirolimus	Completed	lung /other solid tumor/ lymphoma	Metastatic or unresectable tumor	Temsirolimus	Metformin MTD in combination with Temsirolimus
**Biomarker Studies**	NCT02431676	Randomized 3 Arm	Survivorship Promotion In Reducing IGF-1 Trial (SPIRIT)	Sidney Kimmel Comprehensive Cancer Center	effect of metformin compare to 2 other behavioral techniques of wieght loss on IGF-1 level and IGF1 level : IGFBP3 ratio	Active and Recruiting	solid tumor including Lung Cancer	Women with solid tumors BMI > 25kg/m2 WT < 400 lb post curative therapy	Behavioral: Coach Directed Behavioral Weight Loss Behavioral: Self-control weight loss	Self-Directed Weight Loss Coach-Directed Weight Loss Metformin
	NCT02145559	Phase I Randomized	A Pharmacodynamic Study of Sirolimus and Metformin in Patients With Advanced Solid Tumors	University of Chicago	evaluate the pharmacodynamic markers of dual sirolimus and metformin therapy	Active NOT Recruiting	mutliple	Advanced Solid Tumors	Sirolimus	Investigating effects on peripheral T cell levels of P-p70S6k, P-4EBP1, P-Akt

#### Prevention studies

A study from Mayo Clinic (NCT01717482) investigates the feasibility to pursue secondary prevention studies with metformin in Stage IA-IIIA resected NSCLC (Table 3). Patients randomized to metformin vs none, and rates of randomization, accrual and tissue collection are observed.

#### In combination with Chemo-RT

An observational study from Maastricht monitors use of metformin and insulin and induced toxicity in diabetic and control patients with locally advanced NSCLC treated with chemo-RT. Phase II clinical trials investigate metformin as a Chemo-RT-sensitizer and consolidation therapy in locally advanced NSCLC.

The NSABP-RTOG-GOG (NRG) LU001 trial (NCT02186847), is an US NCI CTEP funded study that investigates metformin as a sensitizer to chemotherapy and RT in stage III NSCLC through addition of metformin only during cytotoxic therapy. This includes concurrent Chemo-RT for 6 weeks followed by 6 weeks of consolidation chemotherapy treatment. Chemotherapy, in both the concurrent and the consolidation phase of this study, is the widely used carboplatin-paclitaxel doublet, while patients receive standard chest RT of 60Gy in 30 fractions. Metformin dose is escalated over 2 weeks from 1000mg to 2000mg daily, at which point, is combined with cytotoxic therapy and remains constant for the duration of cytotoxic therapy. This study completed accrual at the end of 2016. A total of 168 patients were accrued. Sample size was designed to detect a 15% improvement in PFS, with the addition of metformin, at 12 months. The first results of this trial may be available by the end of 2017.

An additional, multi-center phase II study that runs in Canada through the Ontario Clinical Oncology Group (OCOG), ALMERA (NCT02115464), examines the potential of metformin to offer both chemo-radio-sensitization as well as consolidation therapy as a single agent following cytotoxic therapy. A total of 98 patients will be randomized in this screening trial to standard Chemo-RT with or without treatment with 2000mg of metformin, which in this study is delivered during cytotoxic Chemo-RT (2 cycles of cisplatin-based chemotherapy, concurrent with chest RT of 63 Gy in 30 fractions) but continues also as consolidation therapy for a total of 12 months. Primary outcome is 12 month PFS. When completed NRG-LU001 and OCOG-ALMERA will provide the first randomized evidence on the potential of metformin to improve the outcomes of standard cytotoxic therapy in locally advanced NSCLC.

#### Metformin with RT alone

A randomized, placebo control, phase II study at MD Anderson Cancer Center investigates metformin in combination with RT alone in inoperable early stage NSCLC patients, treated with stereotactic RT. The primary objectives in this study are tumor response by RECIST criteria and by 18F-PET SUV response (NCT02285855).

#### In combination with chemotherapy

Three studies examine metformin in stage III-IV NSCLC in combination with chemotherapy: one in combination with carbohydrate restriction (NCT02254512), a second with anti-EGFR (Gefitinib: NCT01997775) and a third with anti-angiogenic (Bevacizumab: NCT01578551) targeted therapy, having disease control (PFS) or biomarker (IL6) outcomes.

#### In combination with targeted therapy

Two studies investigate metformin in combination with targeted therapy alone; a phase II study with anti-EGFR (gefitinib: NCT01864681) having PFS as primary outcome and a phase I with anti-mTOR (temsirolimus: NCT00659568) looking for the maximum tolerated dose (MTD) for metformin in this combination.

#### Biomarker studies

Finally, two additional studies examine biomarkers in response to metformin. A three arm study compares metformin to self-directed or coach-directed weight loss and examines effects on IGF-I and IGF binding protein 3 (IGFBP3) levels (NCT02431676) and a second combines metformin with sirolimus and examines effects on the phosphorylation levels of p70^S6k^, 4EBP1 and Akt in peripheral blood T-cells (NCT02145559).

#### Biomarkers for patient selection

To date, there are no established predictive biomarkers of metformin sensitivity in any tumor site. p53, LKB1 and K-Ras status have been proposed as markers of metformin action but, as discussed, pre-clinical studies remain divided on this issue with evidence suggesting that metformin has wide anti-tumor activity independent of the expression levels or mutation status of these signal mediators. It is essential that prospective studies with metformin in lung cancer pursue systematic biospecimen collection to allow investigation of predictive biomarkers of metformin response.

### Investigating metformin in diabetics with lung cancer

A large number of lung cancer patients are diabetic. Some of these patients are already treated with metformin but many are treated with insulin or other hypoglycemic agents alone. For methodological reasons, it has not been possible to include diabetics in the majority of on-going lung cancer clinical trials with metformin. Since, retrospective evidence suggests potential improvement of lung cancer outcomes with metformin treatment for diabetics, efforts should be made to include such patients in future phase III trials. Although, it is very challenging to introduce new anti-diabetic agents in diabetics being prepared for cytotoxic therapy, this may be possible with appropriate supervision by diabetes care teams and introduction of proper stratification criteria in the phase III trial design. Alternatively, prospective studies in diabetics alone could be considered.

## CONCLUSIONS

NSCLC requires urgently effective and well-tolerated chemo-/radio-sensitization therapy. The work discussed here shows that targeting tumor metabolism to enhance the response of NSCLC to cytotoxic therapy is a promising concept. Metformin's ability to activate AMPK and inhibit survival and resistance to chemotherapy and radiation indicates a capacity to compete well with other novel therapeutics. The molecular profile of metformin sensitivity is yet to be determined. K-Ras mutations and loss of LKB1 or p53 need to be investigated further as potential predictive biomarkers. The evidence of chemo- and radio-sensitizing activity and its excellent tolerability allowed metformin to progress rapidly into clinical trials in NSCLC. The on-going randomized studies will begin reporting results in the next 1-2 years. If positive, these studies will assist in rational design of biomarker work and phase III trials.
